# Supplementary material for: Distribution of Pesticides and Polychlorinated Biphenyls in Food of Animal Origin in Croatia
Source: Foods. 2024 Feb 8;13(4):528. doi: 10.3390/foods13040528 (PMC10887917; doi:10.3390/foods13040528)
Supplement: Supplementary file 1 [file foods-13-00528-s001.zip › foods-2845720-supplementary.pdf]

foods

Supplementary for:

Distribution of Pesticides and Polychlorinated Biphenyls in Food of Animal Origin in Croatia

Maja Đokić [1], Tamara Nekić [1], Ivana Varenina [1], Ines Varga [1], Božica Solomun Kolanović [1], Marija Sedak [1], Bruno Čalopek [1], Ivana Kmetič [2], Teuta Murati [2], Darija Vratarić [3] and Nina Bilandžić [1,*]

[1] Laboratory for Residue Control, Department of Veterinary Public Health, Croatian Veterinary Institute, Savska Cesta 143, 10000 Zagreb, Croatia; dokic@veinst.hr (M.Đ.); tamara.nekic@gmail.com (T.N.); varenina@veinst.hr (I.V.); varga@veinst.hr (I.V.); solomun@veinst.hr (B.S.K.); sedak@veinst.hr (M.S.); calopek@veinst.hr (B.Č.)

[2] Laboratory for Toxicology, Faculty of Food Technology and Biotechnology, University of Zagreb, Pierottijeva 8, 10000 Zagreb, Croatia; ikmetic@pbf.hr (I.K.); teuta.murati@pbf.unizg.hr (T.M.)

[3] Veterinary and Food Safety Directorate, Ministry of Agriculture of Republic of Croatia, Planinska 2a, 10000 Zagreb, Croatia; darija.vrataric@mps.hr

* Correspondence: bilandzic@veinst.hr; Tel.: +385-1-6123601

Table S1. GC-MS/MS ion transitions and collision energies (CE).

Compound	Rt (min)	Ion precursor; ion product 1 (m/z)	CE1 (eV)	Ion precursor; ion product 2 (m/z)	CE2 (eV)	Ion precursor; ion product 3 (m/z)	CE3 (eV)
**Organochlorine pesticides (OCPs)**							
Aldrin	23.22	262.8; 193.1	40	262.8; 191.1	40		
Chlordane, cis-	26.68	374.7; 266.0	27	374.7; 302.9	10		
Chlordane, trans-	26.09	374.8; 266.0	30	374.8; 268	22		
DDD- p,p'	28.95	234.8; 165.1	27	234.8; 199.1	20		
DDE- p,p'	27.51	246.0; 175.2	40	246; 176.1	10		
DDT -o,p'	29.06	234.9; 165.0	27	234.9; 199.1	17		
DDT -p,p'	30.13	234.9; 165.1	25	234.9; 199	20		
Dieldrin	27.55	262.7; 192.9	37	262.7; 191	35		
Endosulfan, alpha-	26.58	240.9; 206.1	15	240.9; 171	30		
Endosulfan, beta-	28.66	241.0; 206.0	15	238.8; 204	15	195; 159	5
Endosulfansulfate	30.03	271.9; 236.9	10	271.9; 116.9	40		
Endrin	28.33	262.7; 193.1	35	262.7; 191.1	35		
HCH, alpha-	15.78	218.9; 183.0	7	218.9; 181	7		
HCH, beta-	17.12	218.9; 183.0	6	218.9; 180.8	6		
HCH, gamma- /Lindan	17.46	218.9; 183.0	5	218.9; 181.0	5		
Heptachlor	21.35	271.6; 237.0	15	273.7; 239	15		
Heptachlorepoxid, egzo-	25.10	352.8; 263.0	20	352.8; 281.9	20		
Heptachlorepoxid, endo-	25.29	252.8; 183.1	40	252.8; 181.2	40		
Hexachlorobenzene	16.17	283.9; 213.9	32	283.9; 248.9	32		
Oxychlordane	25.10	386.8; 262.8	14	388.8; 263	14		
Pentachloroaniline	19.94	264.7; 194.0	28	264.7; 203	28		
Pirimiphos-methyl	22.73	290.0; 124.9	25	290; 151	20		
Quintozene	17.72	236.9; 118.9	30	236.9; 142.7	30		
**Organophosphorous pesticides (OPPs)**							
Azinphos-ethyl	33.85	160.0; 132.0	0	132.; 104.0	4	159.9; 104.9	12
Bromophos-ethyl	26.26	358.8; 303.0	15	358.8; 330.9	5		
Carbophenothion	29.78	156.9; 74.9	40	156.9; 121.1	25		
Chlorfenvinphos	25.54	266.9; 159.0	15	266.9; 81	30		
Chlorobenzilate	28.64	250.8; 139.1	15	250.8; 111.1	37		
Chlorpyrifos	23.68	314.0; 258.1	7	314; 286.1	5		

Chlorpyrifos-methyl	21.00	285.7; 93.0	20	287.7; 93.0	20		
Diazinon	18.43	199.0; 92.9	18	199; 135,1	10		
Dichlorvos	7.37	109.0; 78.7	5	184.9; 93	10		
Ethion	29.12	230.8; 128.9	25	230.8, 175	10		
Fenchlorphos	21.83	284.7; 269.8	15	284.7; 93	30		
Fenchlorphos-oxon	21.80	262.0; 109	30	262.0; 121	30		
Fenithrotion	22.57	276.8; 260.0	5	277; 125.1	20	277; 109.1	20
Fenthion	19.30	278.0; 109.1	20	278.0; 124.9	20		
Malathion	23.17	173.0; 98.8	15	173.0; 117.2	10	158.0; 125.0	8
Methidathion	26.18	145.0; 85.0	5	145; 58.1	15	302; 85	16
Methoxychlor	31.82	226.9; 169.1	27	226.9; 141.0	40		
Mevinphos	9.80	127.0; 109.1	11	192; 127	11		
Paraoxon-metyl	14.69	230.0; 200.1	5	230.0; 136.1	5		
Parathion-ethyl	23.70	291.0; 109.0	10	291.0; 81.0	25		
Parathion-methyl	21.00	262.8; 109.0	9	263; 246	2	262.8; 79.1	30
Pirimiphos-methyl	18.45	290.0; 124.9	25	290.0; 151.0	20		
Profenofos	27.39	208.0; 63.1	44	338.9; 269.0	12		
Propetamphos	17.77	138.2; 109.9	5	138.2; 64.2	15		
Pyrazophos	33.70	220.9; 193.1	10	220.9; 149.1			
Tetrachlorvinphos	26.59	330.8; 109.0	20	330.8; 79.0	27		
Triazophos	29.54	161.0; 134.0	5	161.0; 106.0	10	257.0; 162.0	5
**Pyrethroids**							
Allethrin	25.60	123.0; 81.2	7	123; 79.1	22		
Bifenthrin	31.68	180.9; 166.1	12	180.9; 165.2	30		
Cyfluthrin	35.89	162.9; 91.1	15	162.9; 127.1	5		
Cypermethrin	36.64	180.9; 152.1	28	180.9; 127.1	30		
Deltamethrin	41.89	180.9; 152.2	25	253.0; 93.0	20		
Fenpropathrin	31.87	180.9; 152.1	27	181.1; 127.1	35		
Fenvalerate	39.24	166.9; 125.1	10	125.1; 89.1	25		
Permethrin	34.89	183.1; 168.1	15	183.1; 153.1	15	183; 115.2	25
Resmethrin	28.05	123.0; 81.4	10	123.0; 95.0	10		
Tetramethrin	31.67	164.0; 107.3	12	164; 135.1	10		
**Carbamates**							
Carbaryl	21.21	143.9; 115.1	28	143.9; 116.2	13		
Carbofuran	13.15	164.0; 103.0	25	164.0; 149.1	10		
Furathiocarb	32.49	163.0; 107.0	10	163; 77	30	164; 149.2	10
Pirimicarb	19.83	238.0; 166.2	7	166.2; 95.9	15		
**Polychorinated biphenyls (PCBs)**							
PCB 28	20.52	255.8, 186.1	28	257.8; 186	28		
PCB 52	22.48	291.9; 222.0	30	291.9; 220	30		

PCB 101	26.42	325.8; 256.1	39	325.8; 291	12		
PCB 118	28.67	325.7; 256.0	30	325.7; 254	27		
PCB 138	30.27	359.7; 289.9	30	359.7; 324.9	15		
PCB 153	29.40	359.7; 289.9	30	359.7; 287.9	30		
PCB 180	32.23	395.6; 325.9	30	395.6; 360.9	15		

Rt-retention time; CE-collision energy

Table S2. The average values of LOQ, precision, linearity, recovery and RSD in blank fat samples.

**Compound**	**LOQ[a] (µg/kg)**	**Recovery (%)**	**Linearity range**	**RSD[b] (%)**
**Organochlorine pesticides (OCPs)**				
Aldrin	2	82.56	2-250	7.78
Chlordane, cis-	2	95.72	2-250	12.45
Chlordane, trans-	2	80.98	2-250	10.90
Chlorobenzilate	5	89.09	5-250	8.76
DDD- p,p'	1	88.70	1-250	14.09
DDE- p,p'	1	92.65	1-250	12.76
DDT- o,p'	1	94.70	1-250	10.90
DDT- p,p'	1	92.93	1-250	9.87
Dieldrin	2	83.58	2-250	12.78
Endosulfan, alpha-	2	88.04	2-250	13.90
Endosulfan, beta-	2	93.17	2-250	12.07
Endosulfansulfate	2	98.07	2-250	10.65
Endrin	2	93.53	2-250	8.01
HCH, alpha-	1	97.27	1-250	6.86
HCH, beta-	1	94.78	1-250	7.79
HCH, gamma-/Lindan	1	86.45	1-250	5.67
Heptachlor	2	84.33	2-250	7.98
Heptachlorepoxid, egzo-	2	85.07	2-250	4.98
Heptachlorepoxid, endo-	2	83.09	2-250	7.63
Hexachlorobenzene	1	96.45	1-250	5.78
Methoxychlor	1	107.66	1-250	13.45
Pentachloroaniline	1	87.31	1-250	11.89
Quintozene	2	104.03	2-250	13.69
**Organophosphorous pesticides (OPPs)**				
Azinphos-ethyl	5	91.34	5-250	11.23
Bromophos-ethyl	1	83.56	1-250	15.09

Carbophenothion	5	85.78	5-250	13.54
Chlorfenvinphos	1	103.76	1-250	13.89
Chlorpyrifos	1	104.67	1-250	6.90
Chlorpyrifos-methyl	1	108.45	1-250	9.90
Diazinon	2	83.17	2-250	9.09
Dichlorvos	2	81.14	2-250	8.56
Ethion	1	107.70	1-250	4.86
Fenchlorphos	1	99.08	1-250	8.55
Fenithrotion	1	117.76	1-250	7.45
Fenthion	1	82.63	1-250	13.65
Malaoxon	2	96.69	2-250	18.90
Malathion	1	105.45	1-250	17.87
Methidathion	1	90.97	1-250	15.76
Mevinphos	1	82.43	1-250	17.66
Paraoxon-metyl	2	83.56	2-250	10.43
Parathion-ethyl	2	97.74	2-250	12.70
Parathion-methyl	5	102.88	5-250	10.87
Pirimiphos-methyl	1	93.67	1-250	6.78
Profenofos	2	97.69	2-250	9.65
Propetamphos	1	105.58	1-250	4.54
Tetrachlorvinphos	1	91.78	1-250	8.91
Triazophos	1	104.87	1-250	13.65
**Pyrethroids**				
Allethrin	10	80.96	10-250	8.90
Bifenthrin	1	86.87	1-250	14.78
Cyfluthrin	10	92.66	10-250	8.45
Cypermethrin	10	96.87	10-250	13.87
Deltamethrin	10	90.09	10-250	5.76
Fenpropathrin	1	110.75	1-250	9.01
Fenvalerate	10	104.54	10-250	12.99
Permethrin	5	86.09	5-250	13.90
Resmethrin	10	81.09	10-250	13.56
Tetramethrin	1	95.09	1-250	14.94
**Carbamates**				
Carbaryl	2	86.78	2-250	11.76
Carbofuran	2	83.01	2-250	12.78
Furathiocarb	5	107.76	5-250	18.71
Pirimicarb	5	82.27	2-250	8.98
**Polychorobiphenyls (PCBs)**				
PCB 28	0.5	104.65	0.5-250	12.89

PCB 52	0.5	84.65	0.5-250	11.76
PCB 101	0.5	87.97	0.5-250	9.76
PCB 118	0.5	106.85	0.5-250	6.57
PCB 138	1	108.56	1-250	5.90
PCB 153	0.5	97.54	0.5-250	8.98
PCB 180	1	101.86	1-250	7.12

[a]LOQ-limit of quantification; [b] RSD -precision, in case of repeatability

Table S3. The average values of LOQ, precision, linearity, recovery and RSD in blank meat samples.

**Compound**	**LOQ[a] (µg/kg)**	**Recovery (%)**	**Linearity range**	**RSD[b] (%)**
**Organochlorine pesticides (OCPs)**				
Aldrin	2	81.46	2-200	9.08
Chlordane, cis-	2	86.54	2-200	8.87
Chlordane, trans-	2	86.12	2-200	15.76
Chlorobenzilate	2	87.76	2-200	12.09
DDD- p,p'	2	91.73	2-200	11.77
DDE- p,p'	2	92.37	2-200	7.89
DDT -o,p'	2	90.82	2-200	6.98
DDT -p,p'	2	89.32	2-200	8.64
Dieldrin	2	109.67	2-200	9.09
Endosulfan, alpha-	5	94.78	5-200	7.89
Endosulfan, beta-	5	97.23	5-200	8.50
Endosulfansulfate	5	93.12	5-200	7.71
Endrin	2	105.56	2-200	10.67
HCH, alpha-	2	93.55	2-200	8.79
HCH, beta-	2	106.74	2-200	9.73
HCH, gamma-/Lindan	2	105.45	2-200	11.80
Heptachlor	2	97.42	2-200	12.69
Heptachlorepoxid, egzo-	2	91.87	2-200	5.78
Heptachlorepoxid, endo-	5	89.60	5-200	7.90
Hexachlorobenzene	2	80.76	2-200	13.67
Methoxychlor	2	93.76	2-200	12.70
Pentachloroaniline	2	102.67	2-200	11.23
Quintozene	2	97.67	2-200	11.40
**Organophosphorous pesticides (OPPs)**				
Azinphos-ethyl	2	90.87	2-200	13.65

Bromophos-ethyl	2	92.34	2-200	6.78
Carbophenothion	5	84.23	5-200	4.89
Chlorfenvinphos	2	89.19	2-200	14.55
Chlorpyrifos	2	95.54	2-200	11.87
Chlorpyrifos-methyl	2	97.56	2-200	15.50
Diazinon	2	104.67	2-200	7.45
Dichlorvos	2	107.09	2-200	5.78
Ethion	2	102.56	2-200	11.32
Fenchlorphos	2	86.98	2-200	10.65
Fenthion	1	90.87	1-200	15.49
Fenithrotion	2	91.56	2-200	7.77
Malathion	2	106.66	2-200	17.21
Methidathion	2	110.89	2-200	14.32
Mevinphos	2	102.56	2-200	10.91
Parathion-ethyl	2	111.14	2-200	7.89
Parathion-methyl	2	108.64	2-200	5.12
Pirimiphos-methyl	2	96.77	2-200	17.71
Profenofos	2	91.66	2-200	16.76
Propetamphos	2	104.78	2-200	14.55
Tetrachlorvinphos	2	103.67	2-200	12.67
Triazophos	2	97.82	2-200	9.52
**Pyrethroids**				
Allethrin	5	83.67	5-200	16.54
Bifenthrin	2	107.76	2-200	12.23
Cyfluthrin	5	87.16	5-200	16.88
Cypermethrin	5	89.05	5-200	12.07
Deltamethrin	5	81.45	5-200	8.03
Fenpropathrin	2	107.65	2-200	8.25
Fenvalerate	2	87.67	2-200	3.23
Permethrin	5	91.31	5-200	14.98
Resmethrin	5	88.43	5-200	17.65
Tetramethrin	2	98.88	2-200	11.09
**Carbamates**				
Carbaryl	5	102.34	5-200	17.78
Furathiocarb	5	87.01	5-200	4.78
Pirimicarb	5	105.80	2-200	17.90
**Polychlorobiphenyls (PCBs)**				
PCB 28	2	98.65	2-200	7.86
PCB 52	2	92.54	2-200	4.67
PCB 101	2	101.67	2-200	7.54

PCB 118	2	104.76	2-200	5.76
PCB 138	2	88.76	2-200	8.81
PCB 153	2	87.50	2-200	9.97
PCB 180	2	104.64	2-200	8.90

[a]LOQ-limit of quantification; [b] RSD -precision, in case of repeatability
